# Later one knows better: the over-reporting of short-time work in firm surveys

**DOI:** 10.1186/s12651-022-00312-9

**Published:** 2022-07-05

**Authors:** Christian Kagerl, Malte Schierholz, Bernd Fitzenberger

**Affiliations:** 1 Institute for Employment Research (IAB) and FAU Erlangen-Nürnberg, Regensburger Straße 104, 90478 Nuremberg, Germany; 2 Department of Statistics, Ludwig-Maximilians-Universität München, Ludwigstraße 33, 80539 Munich, Germany; 3 IAB, FAU, IFS, CESifo, IZA, and ROA, Regensburger Straße 104, 90478 Nuremberg, Germany

**Keywords:** Short-time work, Survey evidence, Establishment surveys, Non-response bias, Measurement error, C83, J63, J65

## Abstract

Short-time work (STW) in Germany allows for a lot of flexibility in actual usage. Ex ante, firms notify the Employment Agency about the total number of employees eligible, and, up to the total granted, firms can flexibly choose how many employees actually use STW. In firm-level surveys, which provide timely information on STW in Germany, over-reporting of the number of employees on STW is prevalent. This study explores reasons for STW over-reporting based on a high-frequency and low-cost survey initiated during the Covid-19-pandemic (BeCovid) and a low-frequency and high-cost long-running survey (BP). Merging administrative records on actual use of STW, firms that use STW prove more likely to participate in the BeCovid survey. Multi-establishment firms over-report STW because they tend to report STW for all subfirms. The BP uses more interview time and confirms the over-reporting of STW use in the survey month, while—crucially—the over-reporting drops sharply with a few months of retrospection.

## Introduction

Job retention schemes have been the main instrument used to contain the fallout of the COVID-19 crisis on jobs in most OECD countries. Their use has been unprecedented, with take-up as a share of dependent employment in May 2020 about ten times as high as during the peak of the global financial crisis ...

OECD (2021, *p. 101)*

During the Covid-19 pandemic, the usage of short-time work (STW; in German: *Kurzarbeit*) reached unprecedented levels in Germany. At the peak, in the spring of 2020, roughly six million employees from 600.000 establishments were in STW and it took until August 2021 for the number of employees concerned to fall below one million (Federal Employment Agency 2021). STW, a program which has been in place in Germany for a long time, directly subsidizes hours not worked in times of temporary economic distress of a firm to promote job retention. During the crisis 2008/09 and during the Covid-19 crisis, Germany like many countries in the latter crisis increased the coverage of STW and increased the generosity of the scheme (OECD 2021). STW is the main support system to prevent a rise in unemployment and firm closures.

**Fig. 1:**
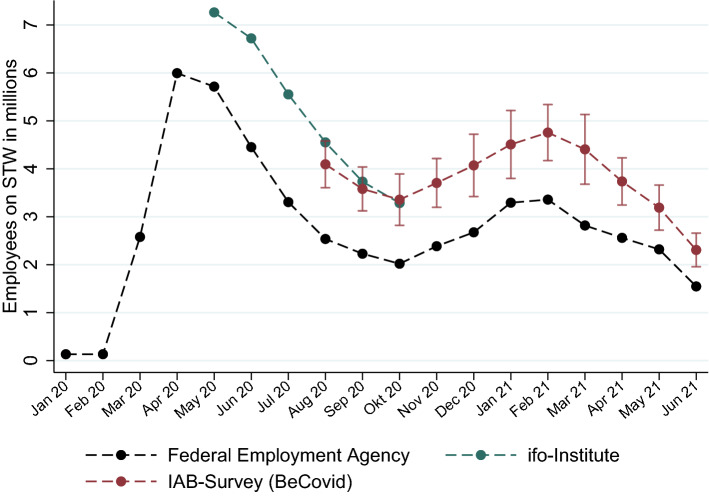
Employees on STW: Administrative Numbers and Unadjusted Estimates from Firm Surveys. *Notes:* The final administrative numbers from the Federal Employment Agency agency are published after a time delay of six months. Numbers from the ifo-Institute are taken from press releases (Link et al. 2020a; Link and Sauer 2020b). Design-based 95% confidence intervals are shown for BeCovid, an IAB firm level survey that started during the Covid-19 pandemic (Backhaus et al. 2021). Raw, unadjusted numbers for BeCovid are shown

STW allows for a lot of flexibility in actual usage by the firm after the approval of the maximum employment level, for which STW is granted for. Administrative records based on actual STW payments to the firm are available only a few months afterwards and it takes six months for the final records to be published by the Federal Employment Agency (Federal Employment Agency 2021; Fitzenberger et al. 2021). Since the number of employees on STW is an important crisis indicator, there was strong demand for real-time data (“now-casting”) of STW usage. This prompted the use of firm level surveys asking about the actual usage of STW in the survey month to provide such information, with the real-time estimates by the ifo-Institute receiving a lot of public attention during the first six months of the Covid-19 crisis (Link et al. 2020a). However, later on, based on administrative records being available six months afterwards, such firm-level survey estimates of STW usage in the survey month were shown to systematically over-report actual STW usage by a sizeable amount. This caused the ifo-Institute to use a downward adjustment of its real-time STW estimated starting in the fall of 2020 based on the over-reporting bias observed for a few months before. Thus, estimates based on firm-level surveys without further corrections do not provide a reliable real-time assessment of STW in times of a fast-moving crises (Link et al. 2020a; Fitzenberger et al. 2021). Figure 1 plots administrative counts of persons in STW by month and shows the comparison to unadjusted totals estimated by monthly firm surveys. The latter show a consistent upward bias relative to the official administrative figures, which are only available with a delay of six months.

The purpose of this paper is to investigate the sources of and reasons for this discrepancy, combining evidence from two different establishment surveys administered by the Institute for Employment Research (IAB)—one with a monthly frequency (BeCovid) and one yearly survey, the Establishment Panel (BP)—which both can be linked to the final administrative records on STW usage by individual firms.

Our analysis shows that a sizeable portion of the overreporting can be traced back to sample selection, such that establishments using STW are much more likely to participate in a survey that focuses on current business conditions. Yet, after reweighting observations to account for this, there still remains a significant difference. Our key novel finding is that this can be traced back to the way STW operates, allowing firms to decide flexibly about the actual use of STW and to claim reimbursement from the Federal Employment Agency with a delay of three months when settling the actual numbers of employees on STW. In fact, the firms’ survey responses become (more) accurate when they are asked about the use of STW in retrospect with a time delay of a few months. Furthermore, we document that establishments that are part of a larger firm containing multiple establishments exhibit less accuracy since one establishment can centrally claim STW for all of a firm’s establishments.

Our results are relevant for the literature investigating the ramifications of STW on indivdual employees and employers and the policy debate on STW (Boeri and Bruecker 2011; OECD 2021), underscoring the importance of measurement error and suggesting some caution when one wants to evaluate STW based on survey data. Our study contributes to the broader literature on measurement error in surveys. For household surveys, this literature typically finds that the reporting error regarding past events increases with the time lag between the event time and the survey time (Bound et al. 2001). In contrast, we find that the error declines with a growing time gap after STW usage because firms know better when they submit the tally to ask for reimbursement by the Federal Employment Agency. Many business surveys ask about information recorded in the firm’s accounting system (Bavdaz 2010) and so a key aspect may be whether the respondent can query the desired accounting information or the interview situation allows forwarding specific questions to colleagues who know better.

This paper is structured as follows: Sect. 2 describes the institutional details of the German STW scheme (*Kurzarbeit*) that are relevant to our investigation. Section 3 analyses the STW differences in a monthly establishment survey, focusing on the potential confounding influences of sample selectivity, record linkage and establishment types which, however, cannot fully explain the observed differences. Section 4 extends the investigation to a yearly survey, comparing the pattern of differences between the two surveys and studying the role of retrospection. Section 5 investigates how accurate STW reporting varies with firm characteristics. Section 6 concludes.

## Institutional background

In the German STW scheme, work lost (i.e. hours not worked) is partially paid for by the unemployment insurance. Different types of STW exist: First, cyclical STW for a limited time when there is less work due to economic reasons or other inevitable events; second, seasonal STW (for specific industries during winter); third, transfer STW (when companies restructure and job cuts are necessary). We focus on the first type, which is the dominant form of STW used almost exclusively during the pandemic, henceforth denoted by STW.

The policy goals of STW are to stabilize employment and firms in order to keep unemployment from surging and to reduce frictions in the recovery after the recession. Only employees subject to social security contributions (roughly 33 million in 2020, i.e., the vast majority of those working) have access to STW, whereas marginal employees or self-employed are not eligible. Access to STW was eased during the pandemic and benefits were increased for those employees on STW for a longer time period (OECD 2021). The (eased) rule involves that 10% of employees in an establishment or department need to have a work loss of at least 10% to qualify for STW. In essence, the German implementation unburdens employers by reducing their wage bill, splitting the costs between the government-backed insurance (that covers a part of the lost hours’ wages) and the employees (who incur losses equal to the part of the wage not covered). For an extended discussion of the types of STW schemes in different countries, their use during the pandemic as well as their potential effects, see OECD (2021).

To use STW, establishments first have to hand in a STW notification (*Anzeige*), detailing the maximum number of employees who might need support and their associated work loss. The establishment pays out the STW benefits to the employees and gets reimbursed afterwards by submitting the tallies (*Anträge/Abrechnungen*) for each month ex post, which detail the actual hours of work lost for each employee. The tallies are checked by the Federal Employment Agency and, if granted, establishments receive the claimed reimbursement. Establishments can submit their claims up to three months after the month in question in which their employees worked less.

A peculiarity concerns the distinction between firms and establishments. Technically, an establishment is a plant at a specified location that pursues a single economic activity, meaning that a firm active at different locations or with various activities may consist of multiple establishments. Yet, for the purposes of the administrative process, one establishment may settle claims for the whole firm (i.e., for different establishments) or multiple departments in the establishment may each submit their own claims. Upon initial approval, establishments can use STW in a flexible way. The approved level specifies the maximum employment level for which STW is granted, but establishments can use less STW than approved, i.e. not every employee listed in the notification has to work less. During the beginning of the pandemic in March and April 2020, 10.7 million employees were listed in the approved notifications, but only 6 million (56%) of those did actually receive support at the peak, with an average work loss for those in STW of roughly 50%.

The number of employees using STW has received a lot of public attention during the pandemic (e.g., the ifo estimates shown in Fig. 1). As pointed out above, administrative data on actual usage of STW as measured by the granted tallies is much lower than the numbers from real-time surveys. We investigate the over-reporting of the actual usage based on establishment (firm) surveys, taking the high-quality administrative data on actual usage (with a time delay of six months) as the benchmark. Only establishments appearing in the administrative data were actually granted the STW reimbursement. Hence, we use these data as the error-free benchmark for the survey data.

## Real-time estimates based on a monthly establishment survey

We first analyze the accuracy of real-time responses regarding employees on STW based on the IAB’s monthly establishment survey BeCovid (“Establishments in the Covid19-Crisis”) that was started to gather information on how establishments were faring during the pandemic. Roughly 2.000 establishments, randomly sampled within strata defined by size (measured by employment) and industry, are asked about various topics in every survey wave, including about STW. Weights are necessary to account for sampling and non-response processes, and weighted results are representative of all privately owned establishments in Germany (for details on the survey, see Backhaus et al. 2021).

The question pertaining to the usage of STW reads *“How many of the employees in your establishment are currently affected by short-time work? An estimate will do.”* If establishments exhibit non-response to this question, a further question is posed concerning the *share of employees* on STW. These questions strike a balance between accuracy and the time budget available for the survey, allowing for estimates from the respondent to minimize non-response. Weighting the responses by the survey’s original weighting scheme produces totals that are consistently too high – as illustrated in Fig. 1.

During the pandemic, administrative data from the Federal Employment Agency based on establishments’ tallies became available, including the number of employees receiving STW benefits for each establishment and month. The establishment identifier being identical in the STW tallies and the sampling frame of the survey allows us to link the survey responses to the administrative data. In this section, we scrutinize whether the weighting approach, record linkage issues, and differential reporting behavior by establishment types can fully explain the observed differences in Fig. 1. While these factors all contribute somewhat, we find that a sizeable part of the difference remains, implying that establishments tend to report figures regarding employees on STW in the survey that are higher than those in their corresponding administrative records.^1^

### Sample selectivity and weighting

**Fig. 2:**
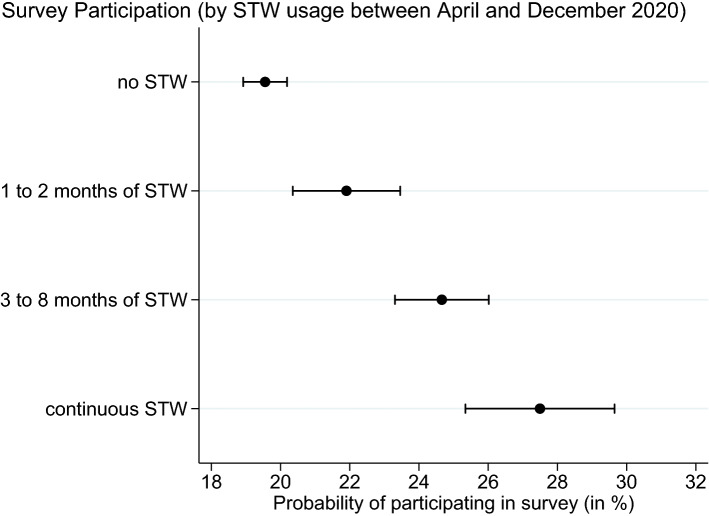
Sample Selectivity. *Notes:* Usage of STW is defined via administrative records. Probability of survey participation comes from dividing all establishments that participated at least once until July 2021 by all establishments that were contacted at least once until July 2021. 95% confidence intervals are shown

We **first** turn to the role played by sample selectivity and weighting by investigating whether the survey participants were using STW disproportionately more often compared to the establishment population we are aiming to represent. Note that the original real-time modelling of non-response behavior to generate sample weights could not account for such a non-response bias because STW usage is unknown at the time. Indeed, as Fig. 2 illustrates, participation depends strongly on how many months in 2020 establishments claimed STW benefits for. While those establishments that never used STW between April and December 2020 roughly had a 20% probability to take part in the survey, this likelihood strongly and significantly increases when claiming STW benefits. For establishments with limited use of STW (1 to 2 months), the value is 22%, while increasing to about 28% for establishments that continuously claimed STW benefits in the nine months from April to December 2020.

Respondents’ willingness to participate in voluntary surveys is known to depend on the survey’s sponsor and its topic. It is highest when respondents want to support an institution they trust on a topic they care about. The IAB-survey BeCovid concerns the impact of the Covid-19 crisis on the labor market and is sponsored by the Federal Employment Agency; both aspects were emphasized in the invitation letter for the survey participants and during the recruitment phase of the interviews. Establishments with STW are likely to be most interested in this topic. Moreover, they already interact with the Federal Employment Agency as they receive money from it. The higher rate of participation among establishments with STW can therefore be attributed to these factors.

**Fig. 3:**
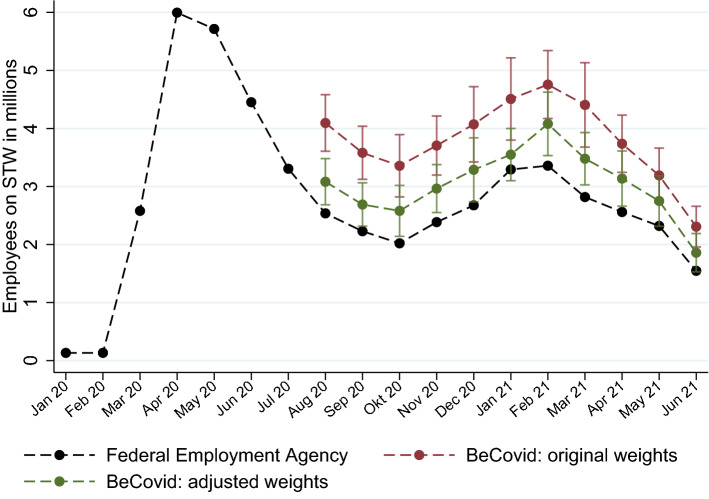
Employees on STW: Administrative Numbers and Estimates With Different Weights. *Notes:* The final administrative numbers from the Federal Employment Agency are available with a time delay of six months. Design-based 95% confidence intervals are shown for both sets of weights

Irrespective of the particular reasons for the higher response rates among establishments on STW, the data allow for adjusting the modelling of the non-response, leading to the construction of a new set of weights for every wave. Figure 3, building on Fig. 1, contrasts the estimates of employees on STW using these adjusted weights with the original ones and with the administrative data on actual tallies.

**Fig. 4:**
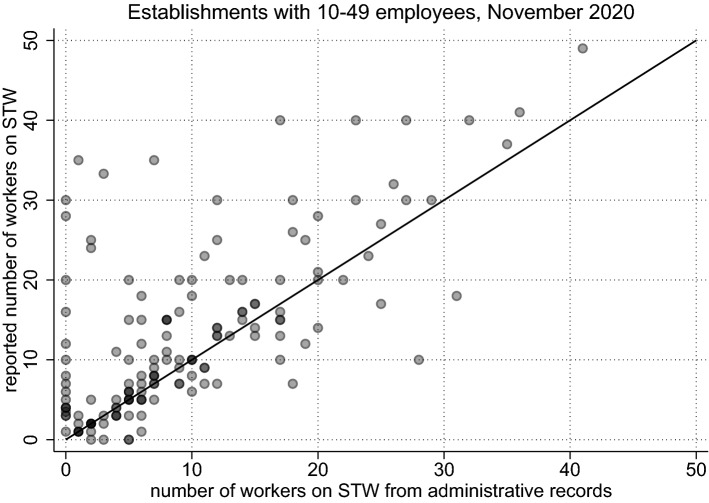
Discrepancy between Survey Responses and Administrative Records. *Notes:* The choice of small to medium-sized establishments is for illustrative purposes. The solid line bisects the plane. Each dot represents the set of establishments with the same observation pair on the x-axis and the y-axis. An increased color intensity of a marker indicates multiple observations with identical pairs

Figure 3 reveals that, while the weighting adjustment considerably reduces the estimated totals, a significant and consistent over-reporting of the final STW numbers still remains. A detailed look at the raw data confirms discrepancies between the survey and the administrative records for the individual establishments, but also reveals that the over-reporting of STW masks considerable heterogeneity. Figure 4 illustrates this point, contrasting administrative records and survey responses for establishments with 10 to 49 employees for the month of November 2020. While the survey responses are on average larger than the administrative records (as can be gleaned from the higher density of points above the bisector), many establishments report accurate numbers and for several establishments the administrative tallies even turn out to be higher than the survey responses.

Next, in a **second step**, we account for the fact that the survey’s sampling frame does not exactly match the universe of establishments in Germany from which the official tallies emanate. A small part of the discrepancies can be explained by such well-understood data collection issues. Table 1, focusing on November 2020 for illustrative purposes (results are very similar for other months), shows that the Federal Employment Agency published a number of 2.386.194 employees on STW, which can be replicated exactly with the administrative data at hand (second column). If we consider only those establishments that the survey could have potentially drawn, the ‘correct’ comparison figure decreases by about 95.000 employees to roughly 2.3 million. Similarly, the benchmark for how many establishments were on STW decreases by about 22.000 to roughly 290.000 as shown in the first column. There are two reasons for these reductions. First, the design of the survey deliberately omitted the public sector, alongside with the tiny sectors households as employers and extraterritorial organizations. Second, establishments with identifiers that do not appear in the data set used for the sampling (which dates from November 30, 2019) are also excluded, involving all establishments started after November 2019.

**Table 1:** Computing Exact Benchmark Numbers (November 2020)

	(1)	(2)
	Establishments	Employees
Officially published number (Federal Empl. Agency)	312.009	2.386.194
Subtracting establishments not in sampling frame	− 22.458	− 95.190
Benchmark number	289.551	2.291.004

*Notes:* See Text for details

Table 2 compares the totals obtained by both sets of weights to the administrative figures. In addition, we quantify the contributions to the totals of establishments who in the survey did not give consent to link their responses with administrative records and had to be excluded for subsequent analyses. Moreover, to assess the quality of the adjustment to the weighting scheme, we include an estimated total which uses the establishment-level numbers from the administrative data in combination with the adjusted weights of the surveyed establishments (columns 3 and 4). The employee benchmark of about 2.3 million is significantly lower (see, in particular, the lower bounds of the 95% confidence intervals) than estimates with the original weights (3.6 million, column 2) and with the adjusted weights (2.9 million, column 4), even though the latter are already reduced by the contributions from establishments we are not allowed to link to administrative records. In relative terms, these numbers imply that 53% of the over-estimation is accounted for by the adjusted weights.

**Table 2:** Comparing Estimated STW Totals to Benchmarks (November 2020)

	Original weights		Adjusted weights			
	Numbers based on Survey Responses				Admin. numbers	
	(1)	(2)	(3)	(4)	(5)	(6)
	Est.	Empl.	Est.	Empl.	Est.	Empl.
Estimated total	450.690	3.646.621	368.100	2.965.178	–	–
no record linkage	− 10.613	− 47.052	− 14.248	− 59.210	–	–
Total (in subset)	440.077	3.599.569	353.852	2.905.968	291.006	2.125.176
95%-CI lower	386.657	3.098.328	306.880	2.499.999	249.757	1.840.340
95%-CI upper	493.496	4.100.810	400.824	3.311.936	332.254	2.410.012
Benchmark	289.551	2.291.004	289.551	2.291.004	289.551	2.291.004

*Notes:* Odd-numbered columns report values for the number of establishments on STW (Est.) and even-numbered columns for the number of employees on STW (Empl.)

Column 6 in Table 2 validates the adjusted weights. The approach uses the surveyed establishments and their weights, but our estimation takes the establishments’ figures from the administrative data instead of the number of employees as reported in the survey. The estimated total is missing in this column because numbers are unavailable for establishments who did not consent to linkage. This analysis constitutes a test of whether the weighting truly makes the sample representative. Reassuringly, the benchmark is well within the confidence interval of the estimated one and the point estimate (2.1 million) lies slightly below, which is to be expected because of those establishments we cannot link to the administrative records. For the number of establishments with STW (columns 1, 3 and 5), the patterns are the same. Hence, the adjusted weights seem valid in making the sample representative with regard to the number of establishments and employees, but can only eliminate roughly half of the survey error (also see Fig. 3). This still leaves a considerable gap.

### Potential record linkage problems

In a **third** step, we decompose the remaining error for the amount of employees into various sources to provide insights into record linkage. Specifically, we consider how much of the discrepancy comes from establishments reporting higher tallies than their records and how much from establishments who report employees on STW but whose establishment identifier does not involve a corresponding administrative record for the respective month. We anticipate such linkage problems because STW claims (used for administrative records) may be settled by an establishment different from where the STW actually occurs (used for survey estimates; also see Sect. 2). For this purpose, Table 3 splits the sample of surveyed establishments (again, from November 2020) into five distinct groups.

The first, and largest, group *A* involves those 389 establishments which report at least one employee on STW for November 2020 and for which there are also administrative records with at least one employee on STW for the month. The row “estimated survey total” in Table 3 denotes the total number of employees estimated with the adjusted weights and using the establishments’ reports from the survey (more than 2.5 million for group *A*) and the row “estimated records total” describes the total obtained by using the surveyed establishments’ administrative STW figures. The following row involves the difference, i.e. the over-reporting effect, amounting to (a highly significant) 463.930 for group *A*, and thus to 59% of the overall (total) difference shown in the last column of Table 3.

Groups *B* and *C* are establishments without administrative STW records in November 2020, despite them reporting a positive amount of employees on STW. On the one hand, there are 52 establishments in group *B* which still have records in at least one other month in 2020. The existence of records in 2020 with their establishment identifiers suggests that group *B* is closely related to group *A*: They might constitute a special case of over-reporting where employers report STW but do not settle claims later on, e.g. because the work loss threshold is not reached. Taken together, the two groups associated with over-reporting (*A* as well as *B*, where record linkage difficulties are unlikely) account for roughly 80% of the total difference. On the other hand, the 23 establishments in group *C* never appear in the administrative data, suggesting potential issues in exactly matching establishments. Also, group *C* shows a sizeable contribution of 23% to the overall difference.

Group *D* consists of establishments reporting zero STW employees but being listed as having received STW, working against the over-reporting effect. However, this group is small and its impact is quite marginal. Finally, the last group *E* of establishments not utilizing STW completes the decomposition, but obviously does not contribute to the observed difference.

**Table 3:** Decomposition of Remaining Survey Error (November 2020)

	(1)	(2)	(3)	(4)	(5)	(6)
	Groups					Total
	*A*	*B*	*C*	*D*	*E*	
*Establishments*						
Reports STW	Yes	Yes	Yes	No	No	
STW record Nov ’20	Yes	No	No	Yes	No	
STW record any month ’20		Yes	No			
Number of establishments	389	52	23	12	1.446	1.922
*Employees on STW*						
Estimated Survey Total	2.557.267	173.497	175.204	0	0	2.905.968
Estimated Records Total	2.093.337	0	0	31.839	0	2.125.176
Difference (S.E.)	463.930	173.497	175.204	− 31.839	0	780.792
	(120.767)	(31.353)	(51.692)	(11.612)	(0)	(135.289)
Contribution to difference	59%	22%	23%	− 4%	0%	100%

*Notes:* Groups of establishments *A*-*E*. *A*: at least one employee on STW based both on survey response and administrative records; *B*: at least one employee on STW based on survey response, no STW administrative record for November 2020 but STW administrative records in at least one other month in 2020; *C*: at least one employee on STW based on survey response, but no STW administrative record in any month in 2020; *D*: no STW report in survey but administrative STW record in November 2020; *E*: no STW records neither in survey nor in administrative data

Even though record linkage problems in matching establishments between the survey and the administrative records exist, we conclude that 80% of the total over-reporting gap is observed in establishments belonging to groups *A* and *B* that are successfully matched between survey data and administrative data.

### Establishment types (single- and multi-plant firms)

As pointed out in Sect. 2, firms settling STW claims for all plants through a single establishment is another potential factor that could explain differences between the survey and the administrative data. While the latter do not include information on whether establishments are single-plant establishments or part of a larger firm, the survey gathered this information. In a fourth step, Table 4 further distinguishes the observed difference, i.e. the over-reporting gap for employees on STW, for groups *A* and *B* from Table 3 by establishment type: single-plant establishments, establishments that are part of larger firm – dividing the latter group by whether an establishment reports being the headquarters or a branch—and those cases with missing information due to item non-response.

**Table 4:** Survey Error by Establishment Type (Groups *A* and *B*, November 2020)

	Establishment type				
	(1)	(2)	(3)	(4)	(5)
	Single plant	Headquarter	Branch	Missing	Total
*Employees on STW*					
Estimated Survey Total	1.691.419	643.989	343.724	51.632	2.730.764
Estimated Records Total	1.273.970	474.762	310.841	33.764	2.093.337
Difference (S.E.)	417.449$$^{***}$$	169.227$$^{**}$$	32.883	17.868	637.427$$^{***}$$
	(94.090)	(78.953)	(21.674)	(10.955)	(124.563)
Ratio of Totals	1.33	1.36	1.11	1.53	1.30
*N* (establishments)	307	86	44	4	441

*Notes:* Considered are establishments from groups *A* and *B*, see Table 3. $$^{*}$$ $$p<0.10$$, $$^{**}$$ $$p<0.05$$, $$^{***}$$ $$p<0.01$$

Columns 2 and 3 in Table 4 show differences in reporting behavior between headquarters and branches in multi-establishment firms, where the ratio of totals is larger in headquarters than in branches. Headquarters over-report significantly (at the 5%-level) with the survey number being 36% larger than the administrative records. However, the administrative records for these establishments might also reflect employees from other establishments of the same firm, i.e. it matters how the firms settle their claims and which numbers they report in the survey.

To explore this issue further, all establishments reporting being part of a larger firm were asked in the survey in November 2020 whether the reported number of employees on STW concerns only the establishment itself or also those of other establishments of the same firm. Subsequently, the establishments were asked to give an updated tally, counting STW only within their own establishment. Based on the updated numbers, the difference for branches in column 3 decreases only marginally from 32.883 to about 30.000. For the headquarters in column 2, the difference drops more strongly to 113.870 (S.E. 77.250, i.e. no longer significantly different from 0), reducing the ratio to 1.24. Thus, while some of the discrepancy is due to headquarters including STW at branch offices in their survey reports, it seems that the administrative records mostly reflect the tallies of the establishment, but this cannot be ascertained as details on the firm structure are absent in the administrative data. However, there is also significant over-reporting of about the same magnitude for single-plant establishments in column 1 as in column 2 for headquarters and in column 5 for all establishments. These findings are quite similar when considering group *A* only.

To sum up, this section has investigated various potential explanations for the over-reporting of employees on STW. First, adjusting the weighting scheme to the higher participation probability of STW establishments cuts the original difference roughly in half. Second, a small part of the remaining difference comes from establishments that never appear in the records in 2020, possibly reflecting record linkage problems. Third, we consider the role played by whether an establishment is a single plant or part of a larger firm. Yet, using validated weights and considering only single-plant establishments without record linkage issues still yields considerable over-reporting. Hence, while the aforementioned factors play an important role, there is convincing evidence for sizeable over-reporting of employees on STW in the survey compared to the administrative records for the same establishments.

## Real-time and retrospective evidence based on an annual survey

The monthly BeCovid survey analyzed above asks for the number of employees on STW numbers during the month when the interview took place (survey month). Since establishments can settle their claims up to three months afterwards (see Sect. 2), they may not know at the time of the interview how many employees in total are actually on STW in the survey month. This means that the real-time numbers reported for the survey month are preliminary in nature. Whether the firm has actually reduced the total labor input (and by how much) can ultimately only be determined after the end of the month. During a pandemic that involved a high degree of uncertainty, establishments’ STW notifications are likely too be on the safe side, i.e. too high. Because the respondent may not distinguish between notifications and actual STW use, he or she is likely to overestimate how many employees will be on STW. For this reason, survey based ‘real-time’ data may necessarily involve an over-reporting bias and the error should be lower in a retrospective survey. To investigate this further, we now turn to the annual IAB Establishment Panel (BP), leveraging that the time in the field for the BP spans several months and that STW numbers of different months were gathered in the various survey months. In this Section, we first ensure comparability between BP and BeCovid and then analyze whether responses become more accurate when the STW month in question, i.e., the month for which the number of employees on STW is asked for, dates some time back.

### Comparability between BeCovid and BP

The IAB Establishment Panel (BP) is a long-running annual survey representative of establishments in Germany (for further information on the data set, see e.g., Ellguth et al. 2014 and Bellmann et al. 2021). One key advantage for our comparison is that the BP is based on the same establishment definition as used by the Federal Employment Agency and draws from the same population of establishments as BeCovid, making (weighted) descriptive statistics neatly comparable. One difference is that BP also includes parts of the public sector, but this is negligible for our analysis as STW was used much less in the public sector. Furthermore, the majority of establishments in the BP are panel establishments that also participated in previous years (i.e., 2019 or before), making the selection on STW usage (as found for BeCovid in Sect. 3.1) much less of an issue.

In the 2020 wave of the BP (which we use), information on the number of employees on STW for different months was elicited. Specifically, establishments were asked: *“Did or does your establishment/office have short-time work since the beginning of the COVID-19 pandemic?”* If this was affirmed, it was asked for which months between March and October 2020 this applied to and *“How many employees were in short-time work? An estimate for all months after the current interview date is sufficient.”* Two main differences to BeCovid (Sect. 3) are noteworthy. First, the use of the question on STW since the pandemic’s start is a filtering device in the BP. Second, a specific number is asked for and, contrary to BeCovid, guesses (estimates) are deemed sufficient for future months after the survey month but not for the current and the previous months. We discuss further differences in the type of questions below.

We use the fact that the BP survey took place in a staggered manner in the summer and fall of 2020 (see, in particular, Sect. 4.2). This means that establishments were interviewed in different months, generating variation in the time gap between survey months and STW months. Unfortunately, it is not known for all establishments when the survey questionnaire was concluded, a limitation due to the multiple survey modes in the BP. No exact survey month can be determined for those establishments that gave responses using questionnaires on paper. We drop these observations because there is no evidence of a selection effect as the usage of STW for establishments with information on the exact date of the survey does not differ from those without. Further restrictions for the BP sample are, first, that roughly 20% of all surveyed establishments do not give explicit consent for record linkage and, second, that some identifiers associated with establishments from the panel have changed after recruitment and do no longer appear in the population data set. Lastly, in order to ensure comparability across the two surveys and to minimize potential inaccuracies resulting from the establishment identifiers, we focus on establishments which—during the STW months March to October 2020 – belong to the equivalent of Group *A* at least once (i.e., establishments with a positive number of employees in STW in both the survey and the administrative data in at least one month; see Table 3). For these establishments, we include all observations that would be classified as Group *B* (positive reported number but no administrative record for the respective month). In total, after applying all restrictions, this leaves us with 3.663 establishments, covering 16.294 establishment-month observations.

**Fig. 5:**
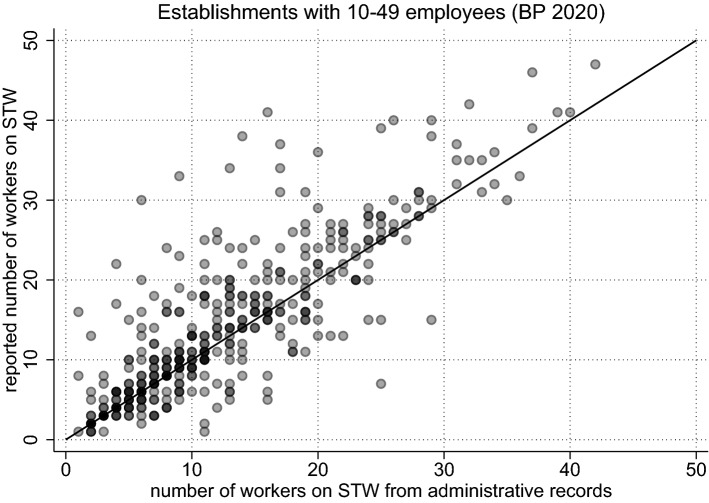
Over-Reporting in the BP (Survey Month = STW Month). *Notes:* The choice of small to medium-sized establishments is for illustrative purposes and for comparison to the BeCovid survey and Fig. 4. Hence, only those establishments are shown where the survey month and the STW month are the same (pooled for the months July until October 2020). Observations are restricted to establishments where both the report and the record are strictly positive (effectively, group *A*). The solid line bisects the plane. An increased color intensity of a marker indicates multiple observations with identical pairs

First, we turn to the comparability of the (raw) data regarding the over-reporting of STW. Figure 5—akin to Fig. 4—plots the reported STW numbers from the BP against the number from the administrative records, for cases where both are positive (effectively, like group *A*). To further ensure comparability to the BeCovid survey, observations are only included if the STW month coincides with the survey month. Due to the BP taking place across different months, Fig. 5 pools establishments surveyed between July and October 2020. Again, as before, the raw data show a higher density of points above the bisector, replicating the STW over-reporting already found for BeCovid.

In general, while STW over-reporting is found in both surveys concerning the concurrent month, the bias is less severe in the BP. While in BeCovid the mean weighted difference (reported survey number minus administrative number) combining groups *A* and *B* amounts to 1.96 (S.E. 0.37) employees in November 2020,^2^ the mean weighted difference in BP is only 1.19 (S.E. 0.24) employees.^3^ Multiple explanations seem likely. First, as mentioned above, the questions differ, especially concerning the fact that BeCovid allows for estimates. This might induce establishments to make guesses based on notifications that turn out to be too high (e.g., instead of more truthfully answering ‘don’t know’). Also, the survey design may play a role. In BeCovid, the interview is restricted to a length of 10 minutes per wave, covering a plethora of topics of which STW is but one. In contrast, the BP is an annual survey with more time available for each question, allowing establishments to locate necessary forms to give as accurate an answer as possible. Nonetheless, there is also considerable STW over-reporting in BP, albeit to a lesser extent than in BeCovid.

### Retrospective evidence

**Fig. 6:**
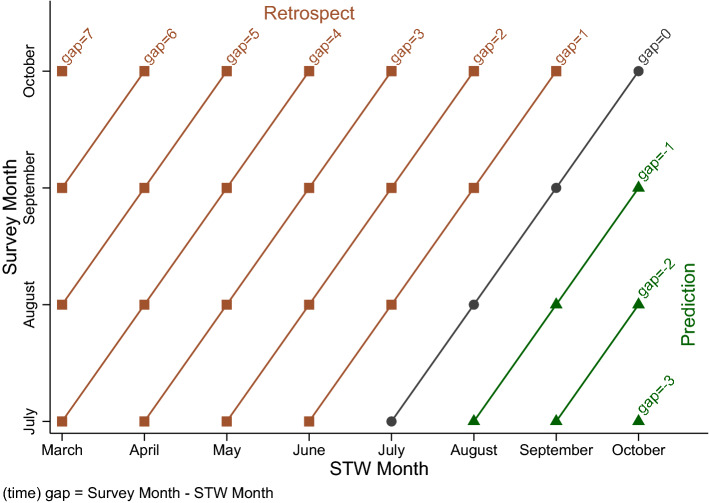
Illustration of Time Gaps Between Survey and STW Month. *Notes:* All months refer to 2020. The illustration shows how the questions on STW in the BP interact with the fieldwork period covering multiple months, creating variation within and between establishments. Example: An establishment is surveyed in July. Its answers on the number of workers on STW for August, September and October are categorized as predictions for the future. Its answer for July (gap=0) is for the same month, i.e. what always is the case in BeCovid. Its answer for June is classified as being one month back, the one for May as two months back, and so forth

Next, we turn to the time gap between the survey month and the STW month. To illustrate this, Fig. 6 plots the range of survey months in BP 2020 (on the vertical axis) against the STW months asked for (horizontal axis). Circles indicate the combinations where STW and survey month are identical (*same month*, gap$=0$), connected by a line. These are the observations already considered in Sect. 4.1, e.g., an establishment reports on STW for August 2020 being asked about it in August 2020. When the survey month lies *before* the STW month (gap$<0$), any report is a prediction (guess) for the future (which we will not focus upon). These cases are marked by triangles and we treat them as one group, irrespective of the time gap. If the survey month lies *after* the STW month (retrospect, square markers), we distinguish by how many months back the STW month is relative to the survey month (gap$>0$). Due to sample size constraints, all observations with a gap of six or larger will be treated as one group. The combinations are categorized according to the time gap by the upward sloping lines in Fig. 6.

Weighted mean differences are reported in Table 5. The first rows of column 1 report some of the aforementioned mean differences if STW month and survey month are the same. Considering reports in retrospect (1 month up to 6+ months in retrospect), the mean weighted difference drops to about 0.2 employees after two to three months, values that are no longer significantly different from zero (standard errors are shown in column 2). Incidentally, if the STW month dates 5 or more months back, the over-reporting seems to increase again. The pattern becomes even clearer when one only considers single-plant firms in the BP. If survey month and STW month are the same, the difference amounts to 0.79 (S.E. 0.22), dropping to a precise 0 (S.E. 0.19) after three months (results not included in Table 5).

As an alternative measure, we consider the share of STW reports that exactly match the number in the administrative record. Figure 8 in the Appendix depicts this share by the time gap between survey month and STW month, separately for single-plant and multi-plant establishments. 39% (not weighted) of single-plant establishments report a number that matches the administrative data if the time gap is zero. This share increases significantly to over 55% after three months.

**Table 5:** Mean Weighted STW Differences by Time Gap between Survey and STW Month

*STW month relative to survey month*	(1)	(2)	(3)
	Mean weighted difference (in employees)	Standard error	Obs.
BeCovid: same month (gap$=0$), Sep ’20	1.89	0.33	383
BeCovid: same month (gap$=0$), Oct ’20	2.17	0.51	344
BeCovid: same month (gap$=0$), Nov ’20	1.96	0.37	441
BP: same month (gap$=0$)	1.19	0.24	1.466
BP: 1 month back (gap$=1$)	0.62	0.22	1.926
BP: 2 months back (gap$=2$)	0.24	0.22	2.255
BP: 3 months back (gap$=3$)	0.23	0.20	2.516
BP: 4 months back (gap$=4$)	0.30	0.18	2.436
BP: 5 months back (gap$=5$)	0.78	0.24	1.719
BP: 6+ months back (gap$\ge 6$)	0.70	0.31	1.068

*Notes:* See text for details

To further investigate whether the time gap can explain differences between reported and administrative figures, we run the following regression utilizing the survey weights:

1 $$\begin{aligned} \Delta _{em} = \alpha + \sum _{x=1}^{6} \beta _x Mx_{em} + \gamma MF_{em} + \delta Mar_m + \psi _e + \varepsilon _{em} , \end{aligned}$$

where $\Delta$ is defined as the reported number minus the administrative one for establishment *e* and calendar month *m*. Positive values of $\Delta$ thus represent over-reporting of employees on STW, while negative values conversely stand for under-reporting. *Mx* denotes dummies that are unity if the difference between STW month and survey month is *x*, e.g. $M2=1$ if the survey month lies two months after the respective STW month (gap$=2$). *M*6 is equal to one if the difference is six or larger. *MF* is equal to one if the number reported in the survey month is a prediction for a future STW month. Survey month and STW month coinciding constitutes the reference category. *Mar* denotes a dummy for March 2020 that we include as a focal point marking the start of the crisis.^4^ At the beginning of the pandemic, the STW support scheme needed to be scaled up first and Fig. 3 shows that the administrative figure concerning employees on STW for March 2020 is much lower than in subsequent months as the first lockdown was instituted only in mid-March. Establishment fixed-effects (FE) are denoted by $\psi _e$. $\varepsilon$ is the error term, and standard errors are clustered at the establishment level. To estimate the effect of retrospective evidence ($\beta$), FE estimates use only the difference *within* establishments over the months in question, which rules out both a potential bias from time-invariant establishment characteristics and from differences in the types of establishments surveyed in different months. However, our results prove robust to dropping the fixed effects, thus alleviating the stated concerns. As ‘intercept’ $\alpha$ in specification (1), we report the average FE (over all establishments), i.e., the FE $\psi _e$ are normalized to sum up to zero. Hence, $\alpha$ can be interpreted as the mean (weighted) difference if survey month and STW month coincide (gap$=0$ and the STW month is not March 2020)—the omitted category.

Table 6 includes estimates for the regression (1) based on four specifications. Starting with column 1, the estimate for $\alpha$ confirms positive average over-reporting of about 1.56 employees if survey month and STW month coincide. The estimates for $\alpha +\beta _x$ ($x \in \{1,\ldots ,5,6+\}$) in column 1 reveal that—relative to the omitted category of survey month and STW month coinciding—a time gap between one and four months reduces the over-reporting considerably. On average, inquiring STW numbers for the month before the survey month reduces over-reporting to 0.85 employees. After four months, over-reporting drops to 0.33 employees—and fluctuates slightly afterwards. Since weighted establishment averages are concerned (with three quarters having less than ten employees), these effects reflect sizeable over-reporting in the aggregate as several hundred thousand establishments were on STW during any month between March and October 2020. The positive $\gamma$-coefficient implies that predictions for the future involve even an stronger upward bias. For March 2020 (as indicated by $\delta$), over-reporting is also higher, possible reasons including recollection error, the first lockdown only taking effect in the middle of the month, and there being a big deluge of STW notifications. For the month of April, no such incremental over-reporting can be discerned (results not reported).

**Table 6:** STW Over-reporting by Time Gap between Survey month and STW month

	(1)	(2)	(3)	(4)
Same month ($$\alpha$$)	1.563$$^{***}$$	1.119$$^{***}$$	3.841$$^{***}$$	1.131$$^{***}$$
	(0.196)	(0.187)	(0.842)	(0.161)
1 month back ($$\alpha + \beta _1$$)	0.851$$^{***}$$	0.520$$^{***}$$	2.581$$^{***}$$	0.565$$^{***}$$
	(0.158)	(0.143)	(0.600)	(0.131)
2 months back ($$\alpha + \beta _2$$)	0.466$$^{***}$$	0.131	2.061$$^{***}$$	0.221$$^{**}$$
	(0.119)	(0.102)	(0.516)	(0.103)
3 months back ($$\alpha + \beta _3$$)	0.555$$^{***}$$	0.099	2.877$$^{***}$$	0.194$$^{**}$$
	(0.116)	(0.094)	(0.676)	(0.098)
4 months back ($$\alpha + \beta _4$$)	0.333$$^{**}$$	− 0.042	1.783$$^{***}$$	0.163$$^{**}$$
	(0.138)	(0.113)	(0.674)	(0.083)
5 months back ($$\alpha + \beta _5$$)	0.503$$^{**}$$	0.020	2.546$$^{***}$$	0.315
	(0.251)	(0.249)	(0.838)	(0.222)
6+ months back ($\alpha + \beta _6$)	0.151	− 0.211	1.633	0.098
	(0.425)	(0.304)	(1.386)	(0.325)
Prediction ($\gamma$)	1.183***	0.938***	2.696*	0.838***
	(0.271)	(0.209)	(1.483)	(0.207)
March 2020 ($\delta$)	1.431***	1.267**	3.008***	1.164***
	(0.271)	(0.237)	(1.057)	(0.224)
Establishment type	All	Single-plant	No single-plant	Single-plant
Unique survey month	No	No	No	Yes
Establishments	3663	2826	760	2664
$N$	16294	12435	3532	11700

*Notes:* Regression estimates for STW differences in equation (1), including FE. Standard errors in parentheses (clustered at establishment level). Group of establishments *A* in BP, see main text for more details. $$^{*}$$ $$p<0.10$$, $$^{**}$$ $$p<0.05$$, $$^{***}$$ $$p<0.01$$

Columns 2 to 4 present sub-sample analyses. Column 2 considers only establishments that report being a single plant and not being part of a larger firm. Over-reporting falls in absolute size and proves insignificant after month 2. The general pattern found in column 1, however, remains unchanged. In column 3, conversely, the sample is restricted to establishments that are part of a larger firm with multiple establishments. Here, over-reporting increases in absolute size. Though decreasing over time, the over-reporting remains large and highly significant even after four months. There is considerably higher statistical uncertainty around these estimates. Column 4 again focuses on single-plant establishments and restricts attention to those with a unique survey month. Due to the fact that the BP has several survey modes, some establishments, e.g,. those filling out a web-based questionnaire on their own, provided survey responses at multiple dates, possibly in different calendar months. For columns 1 to 3, we take the month of the final date—effectively, the submission date—as the survey month. Eliminating establishments without a unique survey month in column 4 does not alter the results in any meaningful way compared to column 2.

**Fig. 7:**
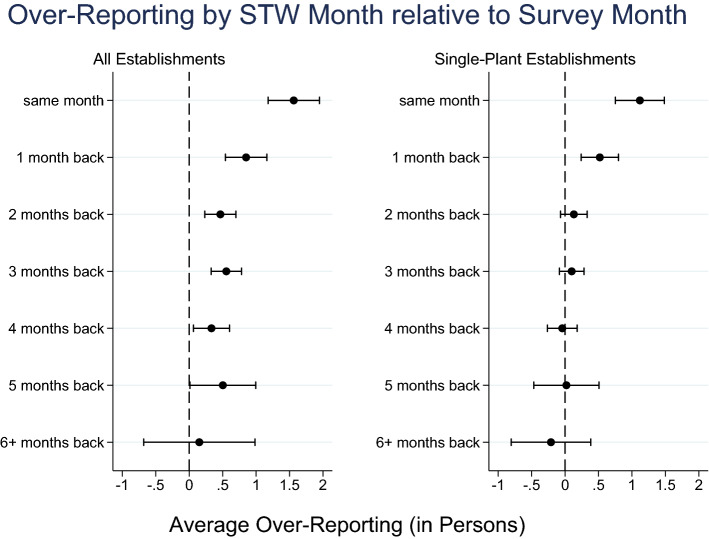
Absolute Weighted Differences in STW Over-reporting. *Notes:* The figure depicts the entries for $\alpha$ and $\alpha +\beta _x$ in columns 1 and 2 in Table 6, including 95%-CIs. These are based on estimates of specification (1) with FE, see main text for further details

**Fig. 8:**
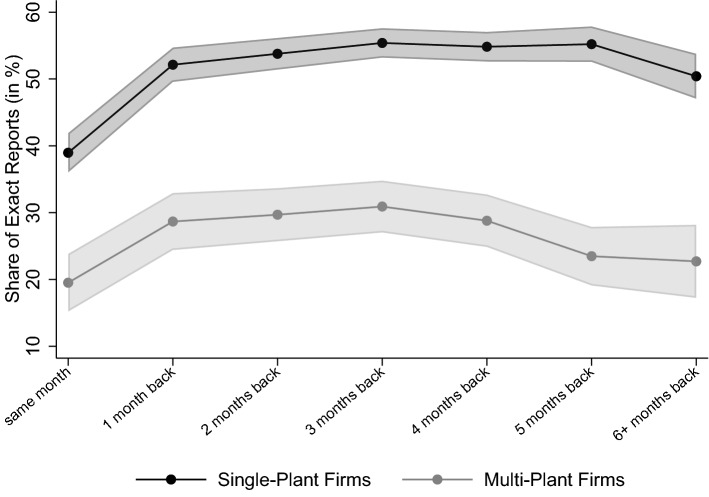
Share of Exact Reports by Discrepancy between STW and Survey Month. *Notes: *A report is ‘exact’ if the number given in the survey exactly matches the number in the administrative record for the respective month. Only establishments where report and record are both strictly positive. Shares and 95%-CIs are are displayed. No weights applied

Taking the estimates in columns 1 and 2 of Table 6, Fig. 7 visualizes the reporting bias by the time gap between survey month and STW month. We focus on the sample of all establishments and the single-plant establishments. We omit the predictions and exclude the estimated effect for March 2020. There is sizeable over-reporting for the same month and this over-reporting is reduced considerable with a growing gap between survey month and STW month. While there remains a significant over-reporting bias for all establishments even five months afterwards, over-reporting becomes negligible and insignificant after two months for single-plant establishments.

Establishments that are part of a multi-plant establishment do not achieve accuracy, even with retrospective responses (see column 3 of Table 6). This most likely reflects that some headquarters report STW for the whole firm and not just for their own establishment or the unit for which they settle the STW claims. Nonetheless, there is a clear pattern of increasing accuracy of survey numbers with a growing time gap. Hence, the very process by which the administrative numbers are generated, with tallies being submitted up to three months afterwards, seems to explain the over-reporting of STW totals. Most establishments need at least the current month to be finished to know the exact numbers needed for submitting the tally. Some establishments may look up their respective tallies during the survey and therefore report the same figure as in the administrative data. Hence, questions posed in retrospect elicit (more) accurate STW numbers.

**Fig. 9:**
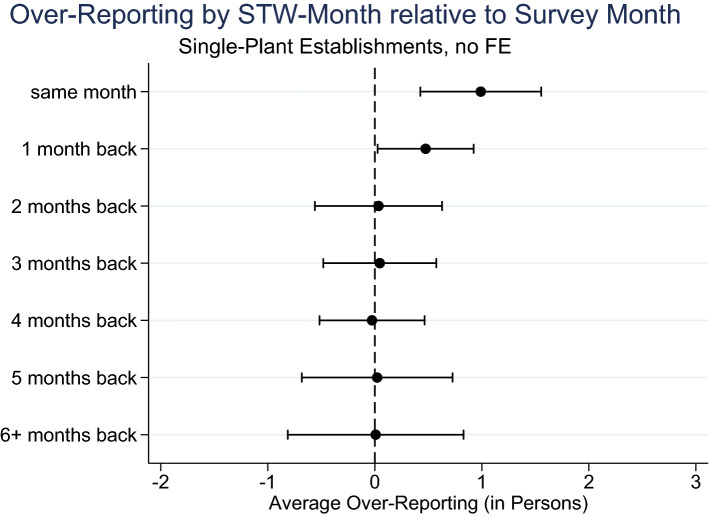
Results without Establishment FE. *Notes:*  Coefficients and 95%-CIs coming from specification (1) without establishment fixed effects are shown. Only single-plant establishments, weights of the BP applied

**Fig. 10:**
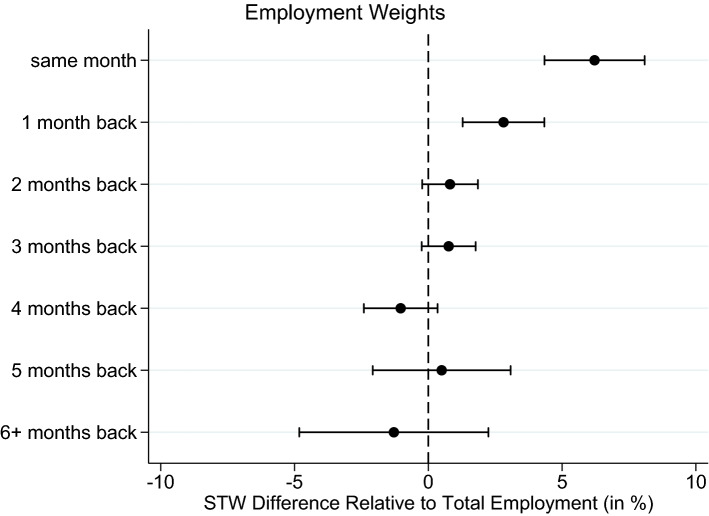
Over-Reporting Results using Relative Differences. *Notes:* Coefficients and 95%-CIs are displayed. Only single-plant establishments, establishment FE are included. As the dependent variable $\frac{\Delta _{em}}{empl_e}$ (see main text for details) has the number of employees liable for social security contributions in the denominator, the more expressive employment weights are obtained by multiplying the establishment weight with the number of employees

We implement a series of robustness checks of the key findings in this Section. Figure 9 in the Appendix presents results without establishment FE, with the findings remaining unchanged. Also, we use relative differences, replacing $\Delta _{em}$ by $\frac{\Delta _{em}}{empl_e}$ in (1), defining $empl_e$ to be the total number of employees subject to social security contributions in an establishment (since only those can receive STW benefits). Results are shown in Fig. 10 in the Appendix. For the same month, over-reporting amounts to roughly 6% of all employees, decreasing to about 3% after one month, and being close to zero and insignificant with a larger time gap.

## Firm-level predictors of misreporting

Our findings so far show that there is considerable over-reporting of employees on STW based on real-time surveys for the current month of STW usage. This over-reporting is reduced considerably for retrospective answers, with the average gap not being significantly different from zero for single-plant establishments after two months. At the same time, there is strong evidence for a large heterogeneity in survey responses both involving over- and under-reporting of employees on STW (as Figs. 4 and 5 attest). Here, we investigate for the BP how such misreporting varies with firm characteristics and whether misreporting is less prevalent when asked in retrospect.

We first look at establishments that have less or equal to ten employees in STW according to administrative records. In real-time reporting (STW month = survey month), 75 percent of very small establishments with at most nine employees report the true number during the survey, but only about 35-40 percent do so among establishments with 10-249 employees (unweighted results). The responses of very small establishments are particularly reliable, most likely because the situation in a very small firm is much easier to assess by the respondent. Nonetheless, due to their very large number, small establishments account for a large portion of the aggregate bias, as Fitzenberger et al. (2021) show. For reporting in retrospect (STW month = survey month - 4) the 75%-number remains basically the same for small establishments, but larger establishments with 10-249 employees and few workers on STW improve their accuracy to about 50-55 percent, suggesting that these establishments are able to check their submitted tallies.

**Table 7:** OLS regression of dummy for misreporting below 10% on firm characteristics

	Real time: STW Month = Survey Month		In retrospect: STW Month = Survey Month - 4	
	(1)	(2)	(3)	(4)
10-49 employees	− 0.365$$^{***}$$	− 0.401$$^{***}$$	− 0.250$$^{***}$$	− 0.266$$^{***}$$
	(0.032)	(0.038)	(0.024)	(0.038)
50-249 employees	− 0.422$$^{***}$$	− 0.460$$^{***}$$	− 0.237$$^{***}$$	− 0.288$$^{***}$$
	(0.035)	(0.041)	(0.027)	(0.042)
250+ employees	− 0.371$$^{***}$$	− 0.336$$^{***}$$	− 0.129$$^{***}$$	− 0.173$$^{***}$$
	(0.054)	(0.067)	(0.044)	(0.066)
Agriculture, Mining, Energy	0.136	0.116	0.061	− 0.251
	(0.166)	(0.182)	(0.105)	(0.220)
Construction	− 0.027	− 0.005	− 0.009	− 0.056
	(0.085)	(0.111)	(0.058)	(0.111)
Wholesale/Retail Trade	0.014	0.030	− 0.018	0.068
	(0.043)	(0.050)	(0.031)	(0.051)
Transport and Storage	− 0.070	− 0.082	− 0.020	0.053
	(0.059)	(0.073)	(0.048)	(0.073)
Accomodation and Food Services	-0.007	0.018	− 0.031	0.014
	(0.053)	(0.059)	(0.037)	(0.059)
Information and Communication	0.099	0.151$$^{*}$$	0.170$$^{***}$$	0.153$$^{**}$$
	(0.065)	(0.080)	(0.056)	(0.078)
Other High-Skilled Services	0.047	0.062	0.008	0.007
	(0.034)	(0.040)	(0.027)	(0.041)
Education, Health, Social Work	0.132$$^{**}$$	0.162$$^{**}$$	$$-$$0.022	$$-$$0.000
	(0.055)	(0.063)	(0.034)	(0.062)
Negatively Affected by Crisis	0.134$^{*}$	0.112	− 0.025	− 0.085
	(0.072)	(0.092)	(0.044)	(0.122)
Single-Plant Establishment	− 0.004	− 0.006	0.113$$^{***}$$	0.075$$^{*}$$
	(0.033)	(0.039)	(0.027)	(0.041)
Survey Month FE	Yes	Yes	Yes	Yes
Number of Establishments	1373	970	2431	970

*Notes:* Based on the BP, establishments from group *A*. Dependent variable equals one if the absolute deviation is at most 10% of the administrative number. Omitted categories: Very small firms (1-9 employees), no single-plant establishment, manufacturing. Standard errors in parentheses, clustered at establishment level. Columns 2 and 4 comprise the exact same establishments. $$^{*}$$ $$p<0.10$$, $$^{**}$$ $$p<0.05$$, $$^{***}$$ $$p<0.01$$

These results are stable when we control for additional covariates. Table 7 includes regression estimates from an OLS regression of a no-misreporting-dummy on establishment characteristics. Misreporting is defined in relative terms, with no misreporting meaning that the absolute difference between the survey response for employees on STW and the administrative number is at most 10% of the administrative number. Note that this definition is equivalent to perfectly accurate reporting for establishments that have less than ten employees in STW. We provide estimates both for the real-time survey responses (columns 1 and 2, STW month=Survey Month) and responses four months in retrospect (columns 3 and 4, STW month=Survey Month-4). Columns 1 and 3 use all available establishment-month observations from group *A*, while columns 2 and 4 are based on only those observations for which both the real-time response and the response in retrospect are available.

A first noteworthy finding in Table 7 concerns the sample differences (column 2 versus 1 and column 4 versus 3). Restricting attention to those cases where both responses are available only has a statistically negligible effect. A negative effect estimate implies that misreporting is more likely for establishments with the corresponding feature (all covariates are dummy variables). Very small establishments (1–9 employees, omitted category) prove much less likely to misreport, even after including further controls.

There are no significant differences in misreporting across industries relative to manufacturing (omitted category), except for Information and Communication showing less misreporting both in real-time and in retrospect and Education, Health, Social Work only in real-time. Being negatively affected by the crisis reduces misreporting in real-time somewhat but not in retrospect. Finally, single-plant establishments misreport significantly less in retrospect compared to other establishments; however, this is not the case for the real-time responses—echoing the findings from Sect. 4.2.

In sum, the findings discussed here augment our earlier evidence. There is a striking difference in misreporting by establishment size, with the smallest establishments being much better in reporting their use of STW. Further, single-plant establishments improve more than others when reporting in retrospect.

## Conclusions

This paper shows sizeable over-reporting of the number of employees on STW based on the IAB’s monthly BeCovid survey and on its annual establishment survey (BP). We leverage the fact that for those establishments consenting to link their survey responses to actual administrative records, which are available with a delay of six months, it is possible to check the validity of the survey answers. While BeCovid only includes real-time survey responses on the use of STW in the survey month, the analysis based on the BP survey also allows to investigate how the over-reporting bias changes for retrospective responses.

There are a number of importing findings. First, in BeCovid establishments using STW are more likely to participate in the survey. This selection bias explains a major part (about half) of the original discrepancy, requiring a weighting scheme that accounts for this. Second, single-plant establishments show a smaller over-reporting bias than establishments being part of a larger firm, most likely because the latter may report STW for all their plants. Without further data on firm networks, however, the dynamics in multi-plant firms are not entirely clear, a possible area for further exploration. Third, the over-reporting bias is less pronounced in the BP, which is a long-running widely used establishment panel survey with an involved interview procedure using multiple survey modes and without hard time limits on answering survey questions. In contrast, BeCovid involves a short, ten-minute, interview to elicit quick responses on the current situation of the establishment in real-time. Fourth, and most importantly, the over-reporting falls considerably for retrospective responses in the BP and, after a time gap of two months, the bias entirely disappears for single-plant establishments, owing to the way the STW scheme operates in Germany. Fifth, we document a strong heterogeneity in the accuracy of survey responses in comparison to the administrative records, suggesting that establishments are quite uncertain about the current use of STW at the time of the survey. We find that the smallest establishments do better in exactly reporting their use of STW and that single-plant establishments improve more than others when reporting in retrospect.

Altogether, our findings suggest the importance of checking carefully real-time estimates based on establishment surveys, especially in a crisis situation. There is strong evidence that real-time estimates of STW usage suffer from a strong over-reporting bias, given sample selection processes and the scheme’s current implementation. Therefore, real-time measurement of STW via a firm survey remains notoriously difficult. Resorting to ad hoc adjustments to account for the bias using aggregate data only (Link and Sauer 2020b) may be difficult to justify without establishment-level evidence on the accuracy of STW reports. At the same time, the predictions of the Federal Employment Agency (Federal Employment Agency 2021) for the final administrative figures, which are available two months after the STW month, have become quite accurate over the course of the Covid-19-crisis. Finally, our investigation also highlights that studies aiming to identify the economic ramifications of STW based on survey data need to take measurement error into consideration.

## Data Availability

Data from BeCovid and the BP are available via the Research Data Centre (FDZ) of the Federal Employment Agency at the Institute for Employment Research. Due to data protection regulations, the administrative records on STW are currently—to the best of our knowledge—only available within the Institute for Employment Research. BP data on when establishments were surveyed are not available via the FDZ and were provided by Susanne Kohaut.

## Footnotes

1 Some of the points in this section have been discussed, in German and targeted to a non-research audience, in the policy report by Fitzenberger et al. (2021).

2 For September and October 2020 the BeCovid values are 1.89 (S.E. 0.33) and 2.17 (S.E. 0.51), respectively.

3 This holds when excluding differences that are smaller than $-200$ or larger than 200. If outliers are not excluded, the BP value is 1.47 (S.E. 0.79) employees, with the difference being driven entirely by 10 out of about 1.500 establishments. Such large differences ($|$difference$|$ $> 200$) almost never occur in the BeCovid survey which includes only few very large establishments, confirming the lower over-reporting in BP.

4 In an augmented specification, we also included a dummy for April 2020 but this made no discernible difference in the results and had no explanatory power.
